# Impact of biofertilisers on iron homeostasis and grain quality in the rice variety Uma under Elevated CO_2_

**DOI:** 10.3389/fpls.2023.1144905

**Published:** 2023-06-22

**Authors:** M. S. P. Bhavya, R. V. Manju, M. M. Viji, S. Roy, K. N. Anith, R. Beena

**Affiliations:** ^1^ Department of Plant Physiology, College of Agriculture, Vellayani, Kerala Agricultural University, Thiruvananthapuram, Kerala, India; ^2^ Department of Agricultural Microbiology, College of Agriculture, Vellayani, Kerala Agricultural University, Thiruvananthapuram, Kerala, India

**Keywords:** elevated (CO2), iron homeostasis, PGPR - plant growth-promoting rhizobacteria, biofertilizers, rice

## Abstract

The diminishing nutritional quality of rice with increasing concentrations of atmospheric CO_2_ is currently a major global concern. The present study was designed with the objective of assessing the impact of biofertilisers on grain quality and iron homeostasis in rice under elevated CO_2_. A completely randomised design with four treatments ([KAU, POP (control), POP+Azolla, POP+PGPR, and POP+AMF]), each replicated three times under ambient and elevated CO_2_ conditions, was followed. The analysed data revealed that yield, grain quality, and iron uptake and translocation were modified in an unfavourable manner under elevated CO_2_, which was reflected in the lower quality and iron content of the grains. The response of iron homeostasis in the experimental plants to the application of biofertilisers, especially plant-growth-promoting rhizobacteria (PGPR), under elevated CO_2_ strongly suggests the possibility of utilising them for designing iron management strategies for achieving higher quality in rice.

## 1 Introduction

Iron (Fe) is one of the most essential micronutrients for plants and humans. In plants, iron is important for life-sustaining processes, such as respiration and photosynthesis. In humans, Fe is essential for the formulation of hemoglobin, DNA synthesis, strengthening the immune system, etc. Despite its importance, iron deficiency is the most prevalent and widespread nutrient deficiency among the global population.

Rice (*Oryza sativa* L.) is the staple food for nearly half of the world’s population and had a global production of more than 740 million tons in the year 2014 (FAOSTAT, 2016). In terms of micronutrient content, polished rice grains have low levels of iron (Johnson, 2012). The regions with the highest rates of poor iron consumption were those where rice dominates the majority of diets. Many people in underdeveloped nations that primarily consume cereals are iron deficient. When grains are milled, Fe concentration is drastically reduced because the Fe-rich portions of the grains are eliminated during the milling process (Trinidad et al., 2009).

Atmospheric CO_2_ is increasing rapidly and is expected to surpass 550 ppm within this century (Lamichaney and Maity, 2021). By the end of the 21st century, the surface air temperature is expected to rise by 1.1°C to 6.4°C because of rising emissions of CO_2_ and other greenhouse gases. This rapid CO_2_-induced temperature rise in the atmosphere negatively impacts the productivity of agricultural crops (Pareek et al., 2020). The temperature range of 30–33°C during flowering, 20–30°C during grain filling, and 15–20°C temperatures at night are favourable for rice growth and development. Higher mean temperatures will impact the growth stages and grain formation in rice (Beena, 2013; Beena et al., 2018; Pravallika et al., 2020). Therefore, global change might undermine the world’s food security and result in significant agricultural losses.

Zhu et al. (2018) reported declines in iron in 18 genetically diverse rice lines under elevated CO_2_. Rice grains grown at elevated CO_2_ have 5.2% lower iron content than those grown at ambient CO_2_ (Myers et al., 2014). Under elevated CO_2_, iron content in rice grains was reduced by nine percent in a study by Jena et al. (2018). Rice grain Fe concentration was lowered by 10.9 percent during polishing (Ujiie et al., 2019).

Iron absorption and accumulation were reduced under elevated CO_2_ because it alters the expression of iron transporter genes, including *OsZIP3*, *OsZIP5*, and IRT-like proteins (Ujiie et al., 2019). As plants use CO_2_ as a substrate for photosynthesis, an increase in atmospheric CO_2_ concentration may promote the accumulation of carbohydrates in plants, which may have the “dilution effect” of lowering Fe content in plant parts, mainly in grains. With elevated CO_2_, elemental re-translocation is affected *via* carbohydrate translocation, which decreases grain Fe content. Increasing atmospheric CO_2_ is predicted to worsen the situation by impacting the uptake and translocation mechanisms of iron. Almost 1.4 billion children and women of childbearing age (59% of the global population) have been predicted to be at risk of increased iron deficiency because of increased atmospheric CO_2_ within the next two decades (Smith et al., 2017), which can lead to severe anemia.

Improved rice yield and quality and increased iron content in rice can be achieved using microorganisms from the rhizosphere or those injected into the soil. This can be linked to the function of biofertilisers in controlling rhizosphere reactions by excreting hormones, plant growth regulators, and siderophores that improve Fe availability. *Azolla* incorporation increases the paddy yield by 8–14% (Yao et al., 2018). It releases plant growth regulators and vitamins that enhance the growth of rice. *Azolla* also solubilises Zn, Fe, and Mn and makes them available to the rice crop. Arbuscular mycorrhizal fungi (AMF) enhance ferric chelate reductase activity under Fe deficiency, which activates the uptake mechanisms of nutrient transporters (Rahman et al., 2020). AMF plants increase the allocation of N and P to rice panicles compared with non-AMF plants during the grain filling stage and the grain yield of rice has been shown to increase by approximately 28% (Zhang et al., 2016).

Plant-growth-promoting rhizobacteria (PGPR) enhance plant growth and are directly involved in increasing the uptake of nitrogen, the synthesis of phytohormones, the solubilization of minerals, such as phosphorus, and the production of siderophores by 17-fold, which chelate iron and make them available to the plant roots (Katiyar and Goel, 2004). The *Bacillus* strains present in PGPR are very strong solubilisers of iron and increase mugineic acid biosynthesis, which enhances Fe chelation capacity and Fe uptake. In a comparative proteomics analysis, PGPR upregulated the nicotianamine synthase 1 (*NAS1*) gene by 100-fold in rice. PGPR also increased ferritin isoforms (*Fer552* and *Fer768*), which increased Fe storage in the grains (Alberton et al., 2020).

As the CO_2_ concentration of the atmosphere is rising day by day, it is of great importance to understand the effect of this elevated CO_2_ on crop growth and development and also on the nutrient status of the crop. The deleterious impact of increasing atmospheric CO_2_ on the quality of cereal grains is already well documented, especially in terms of reducing micronutrient levels (Radha et al., 2023). Anemia due to iron deficiency is becoming a serious global health issue. In this context, efforts have been made to address this issue. The present study was designed to assess the importance of biofertilisers for iron homeostasis under elevated CO_2_ in the most popular variety of rice in Kerala, Uma.

## 2 Materials and methods

### 2.1 Methodology

The experiment was conducted using the open top chamber (OTC) facility at the Department of Plant Physiology, Kerala Agricultural University (KAU), College of Agriculture, Vellayani, situated at 8°5’ N latitude and 76°9’ E longitude at an altitude of 29 m above mean sea level.

Seeds of the rice variety Uma (MO16) were collected from the Integrated Farming Systems Research Station, Karamana, Thiruvananthapuram district, Kerala. Biofertilisers (PGPR and AMF) were collected from the Department of Microbiology, and *Azolla* was collected from the Instructional Farm, Kerala Agricultural University, College of Agriculture, Vellayani.

All the microbial cultures used in the present study were obtained from the culture collection of the Department of Agricultural Microbiology, College of Agriculture, Kerala Agricultural University (KAU). All of them are isolated and characterised at KAU. PGPR mix I contained a consortium of beneficial microorganisms. They include *Azospirillum lipoferum* strain CRT1 (NCBI Accession number DQ438997.1), *Azotobactor chroococcum* strain AAU1013 (NCBI Accession number KF494188.1), *Bacillus megaterium* strain SZN4 (NCBI Accession number EU256396.1), and *Bacillus sporothermodurans* strain CB281428 (NCBI Accession number JX840987.1) (Gopi et al., 2020). The PGPR isolates have been used as efficient plant growth promoters in many crops, including rice (Bal et al., 2013; Liu et al., 2022). Our aim was to investigate whether they could also perform well under elevated CO_2_ and at higher temperatures, and if so, how would they influence iron homeostasis and grain quality in rice.

### 2.2 Nursery

The seeds of rice variety Uma were soaked in distilled water for 24 h and placed in Petri plates for germination. The germinated seeds were grown for 20 days in a tray containing paddy soil.

#### 2.2.1 Inoculation of arbuscular mycorrhizal fungi at the nursery

AMF inoculum (200 g) was applied to 1 m^2^ of a nursery tray. The germinated seeds were grown in the tray for 20 days. The seedlings were then transplanted to the pots treated with AMF in ambient and elevated CO_2_ conditions and were maintained until harvest. The application of nutrients and further maintenance of the experimental plants were carried out as per the recommendations of the Package of Practices (KAU, 2016).

### 2.3 Transplantation

Twenty day-old rice seedlings of uniform height were selected and transplanted with two seedlings per pot. Plants were grown in mud pots with a diameter of 17 cm and a height of 25 cm, and the water level was maintained at a depth of 2 cm. Before transplantation, the pots were treated with the respective biofertilisers.

#### 2.3.1 Soil preparation

The experiment was conducted as a pot study. Each pot was filled with 5 kg of a potting mixture of sandy clay loam soil and farmyard manure at a ratio of 4:1.

#### 2.3.2 Inoculation of *Azolla* and plant-growth-promoting rhizobacteria

*Azolla* (fresh weight of 12 g) was incorporated into pots 5 days before transplantation. Experimental pots treated with *Azolla* were maintained in ambient and elevated CO_2_ conditions, and seedlings were transplanted into the respective pots. PGPR I (20 g) was mixed with 1 kg of FYM. This FYM was used for the preparation of potting mixture in ambient CO_2_ and elevated CO_2_ pots, and seedlings were transplanted into the respective pots. The application of nutrients and further maintenance of the experimental plants were carried out as per the recommendations of the Package of Practices (KAU, 2016).

Composition of the PGPR: *Azotobacter chroococcum, Azospirillum lipoferum, Bacillus megaterium, Bacillus sporothermodurans* (Gopi et al., 2020).

### 2.4 CO_2_ enrichment technique

The main system used for subjecting the plants to an elevated CO_2_ (eCO_2_) environment is an Open Top Chamber, Department of Plant Physiology, College of Agriculture, Vellayani. For the present programme, a CO_2_ concentration of 500 ppm was maintained within this chamber. The Open Top Chamber (OTC) system consists of square chambers that maintain eCO_2_ conditions for experimental studies. The basic structure of the OTC was a metal frame covered with a 200 µm UV poly sheet. The chamber dimensions were 3 × 3 × 3, with a 45° slope a 1 m^2^ opening at the top. CO_2_ was released into the chamber from a CO_2_ cylinder in a controlled manner. The measurement of microclimatic parameters (temperature, humidity, and light) was carried out inside and outside the OTC with the help of sensors on a real-time basis. Light intensity was measured using a LI-COR Model Li-250 light meter.

Ambient CO_2_-grown plants were grown outside the OTC in the field where the average temperature was 32.8°C and humidity was 85%, whereas within the OTC the average temperature was 36.5±8°C and humidity was 60.55%. There was no significant difference in the light intensity values. The light intensity values were 3,116.98 µmol M^2^ s-^1^ in ambient conditions and 2,501.03 mol m^2^ s-^1^.

### 2.5 Observations

#### 2.5.1 Plant growth and yield parameters

##### 2.5.1.1 Number of tilleaccumulation of photosynthates in the shootsrs and productive tillers

The total number of tillers at the active tillering stage and panicle bearing tillers at the time of harvest were counted in each plant.

##### 2.5.1.2 Dry matter production

Plant samples from each treatment were uprooted, washed, sun dried, and then oven dried at 65 ± 5°C until a constant weight was obtained (expressed in g plant^-1^).

##### 2.5.1.3 Straw yield

The harvested straw from each plant was collected separately and dried under the sun for three consecutive days and the weight was expressed in g plant-1.

##### 2.5.1.4 Number of grains per panicle and grain yield

Panicles were hand threshed and total grains per plant were counted. The grains were harvested from each plant separately and dried in the sun to a moisture content of 14% and their weight was recorded and expressed in g plant^-1^.

#### 2.5.2 Quality analysis of grains

##### 2.5.2.1 Estimation of total carbohydrates

The total carbohydrate content was estimated using the Anthrone method (Hodge and Hofreiter, 1962). Powdered grain samples (100 mg) were taken and hydrolysed in a boiling water bath for 3 h with 5 ml of 2.5 N HCl. Sodium carbonate was added until the effervescence ceased, and the volume was made up to 100 ml and centrifuged at 5,000 rpm for 15 min. The supernatant was collected and divided into three aliquots of 0.1 ml, 0.2 ml, and 0.3 ml in volume; these aliquots were then made up to 1 ml with distilled water to generate three concentrations of the supernatant, and 4 ml of anthrone reagent was added. The reaction mixture was heated in a water bath for 8 min and allowed to cool. The colour change from green to dark green was observed and read at 630 nm in a spectrophotometer (Spectronic-200). Glucose was used as a standard (10 mg to 100 mg concentrations) for the estimation of carbohydrates in the leaves.


Calculation:Amount of total carbohydrate=mg of glucose/volume of test sample×100

##### 2.5.2.2 Estimation of total soluble proteins

The total protein content was estimated using the procedure described by Bradford’s method (1976). Bovine serum albumin (BSA) was used as standard solution at different concentrations viz. 20 mg, 40 mg, 60 mg, 80 mg, and 100 mg. Plant samples (500 mg) were digested with 10 ml of phosphate buffer solution (PBS) in a mortar and pestle. The homogenate was centrifuged at 3,000 rpm for 5 min. The supernatant (0.1 ml) was transferred to a volumetric flask and the volume was made up to 3ml with PBS, to which Coomassie Brilliant Blue Dye (5ml) was added; the absorbance was measured at 595 nm using a spectrophotometer (Spectronic-200). The protein content was expressed as mg g^-1^.

##### 2.5.2.3 Estimation of amylose content

Amylose content was determined using the method described by Sadasivam and Manickam (1992). Distilled ethanol (1 ml) and 10 ml of IN NaOH were added to 100 mg of powdered rice sample, which was then incubated overnight; the volume was then made up to 100 ml. The extract (2.5 ml) was taken and approximately 20 ml of distilled water was added along with three drops of phenolphthalein. Then, 0.1 N HC1 was added drop by drop until the pink colour disappeared. To this, l ml of iodine reagent was added and the volume was made up to 50 ml. The intensity of the developed colour was read at 590 nm in a spectrophotometer (Spectronic-200). The amylose present in the sample was estimated from a standard graph prepared using a serial dilution of standard amylose solution and expressed as a percentage.

##### 2.5.2.4 AMF colonization

The roots were cut 15 days after the inoculation of plant-growth-promoting microorganisms in the seedlings. The roots were washed thoroughly in water to remove the soil particles and cut into 1-cm pieces. Then, the root pieces were soaked in freshly prepared 10% KOH solution overnight and boiled in 10% KOH for 1–2 h. After boiling, the pieces were washed in water three times and acidified with 1N HCl for 3 min. The acid was drained and the root pieces were stained with Lactophenol Trypan Blue (LTB) for 10 min (Anith et al., 2011). After 10 min, the root pieces were transferred to lactophenol solution for destaining. The presence of mycelial networks and vesicles were checked for VAM colonization.

AMF colonization studies were performed at the time of harvest. AMF-treated plants under eCO_2_ condition showed 33% colonization at the time of harvest and 30% in plants grown in ambient conditions. **Figure 3** shows the survival and colonization of VAM at the time of harvest.

**Figure 1:**
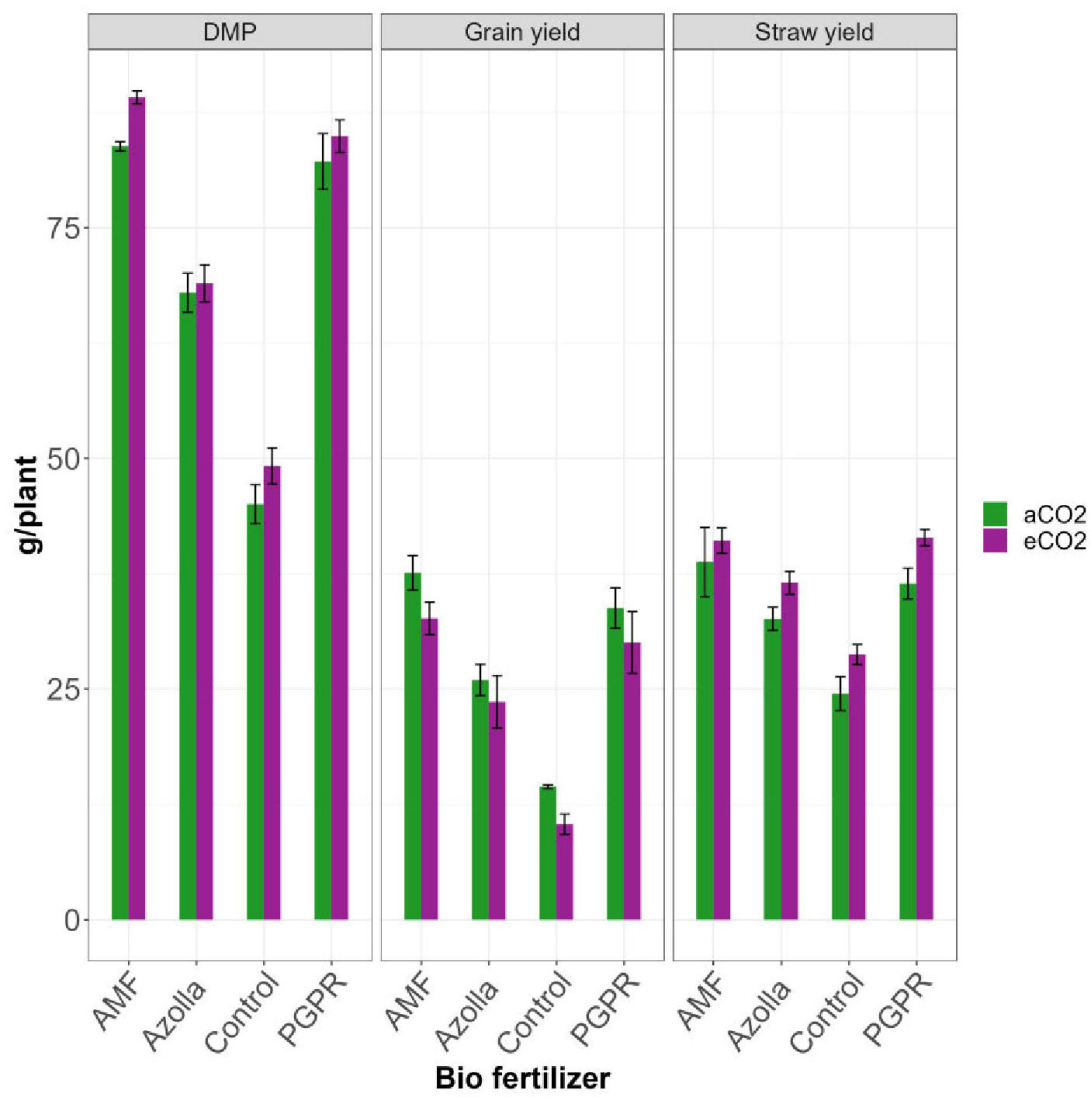
Impact of biofertilizers on dry matter production, straw yield and grain yield under ambient and elevated CO2 conditions.

**Figure 2:**
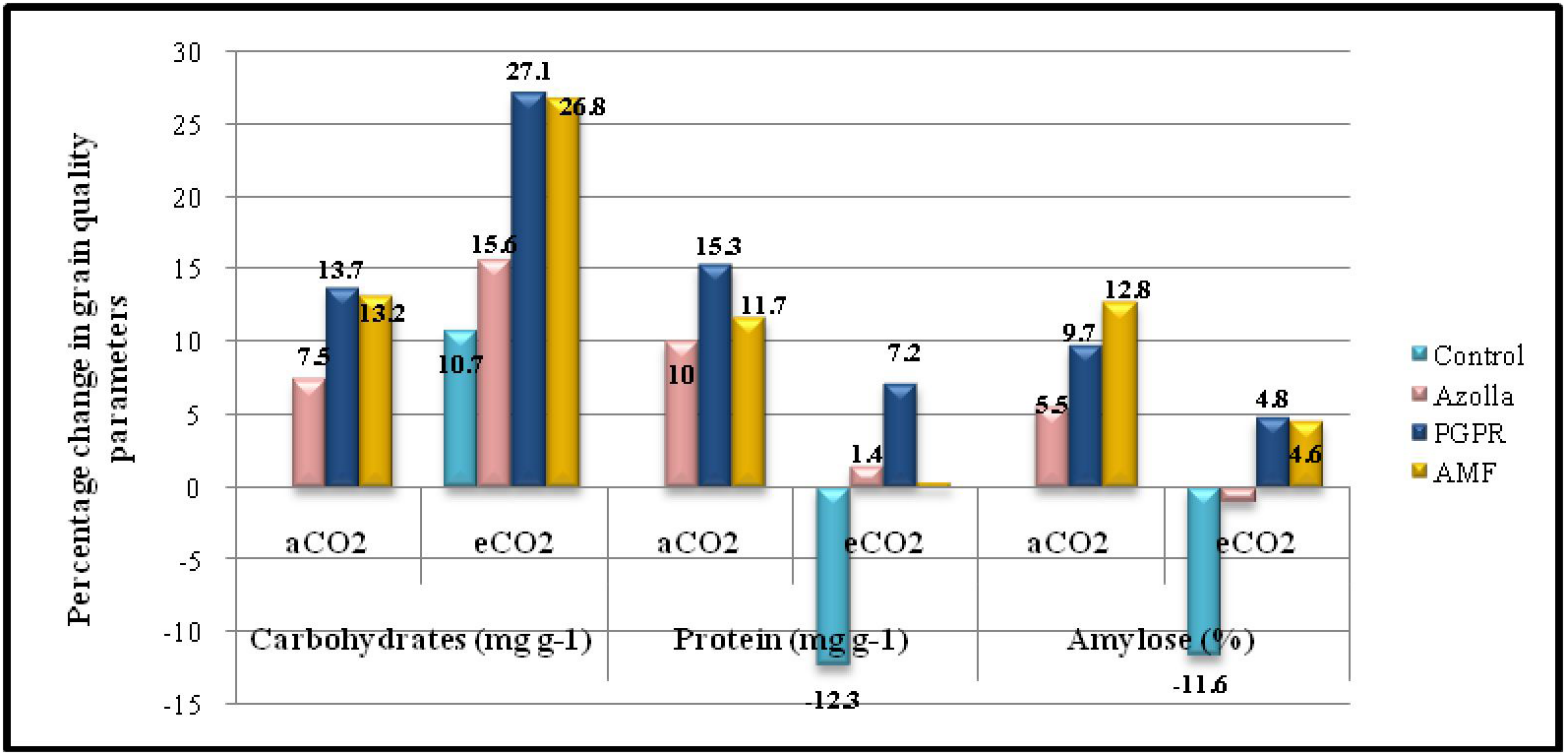
Percentage change in grain quality parameters in comparison with control plants under ambient CO2 condition.

**Figure 3:**
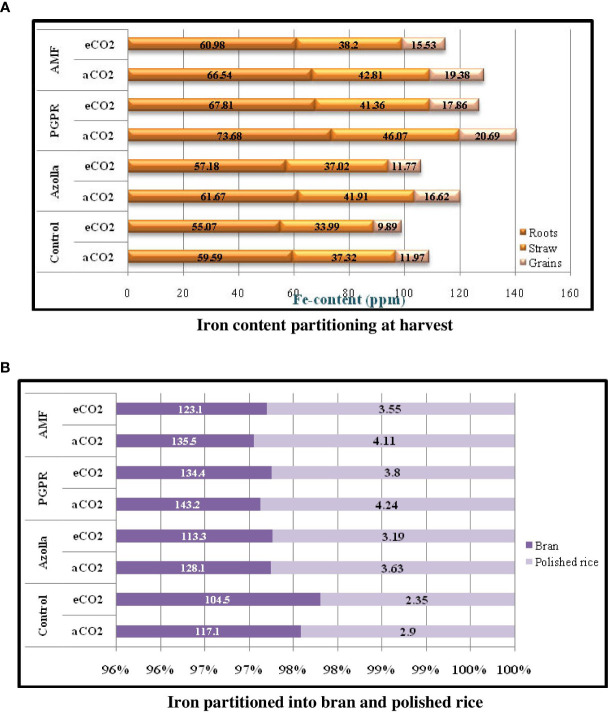
Iron content partitioning in various parts of rice plan (3a) and iron partition in grain (3b) during harvest stage in comparison with control plants under ambient condition.

#### 2.5.3 Estimation of iron content

##### 2.5.3.1 Diacid digestion of plant samples

Plant samples (1 g) were placed in a 100-ml conical flask to which 10 ml of digestion mixture (nitric acid and perchloric acid at a ratio of 9:4) was added, followed by gentle shaking. The conical flask was placed on a hot plate and allowed to boil until the solution became clear and colourless. After cooling, the solution was transferred to a 100-ml volumetric flask and made up to 100 ml.

Iron content was estimated and expressed in μg g^-1^ (or) mg kg^-1^ (or) ppm. Fe content was calculated using an atomic absorption spectrophotometer (PinAAcle 500) (Gitz et al., 2018) at the Department of Soil Science and Agricultural Chemistry, Kerala Agricultural University, College of Agriculture, Vellayani.

##### 2.5.3.2 Calculations


1) Pre-anthesis Fe uptake(kg/ha)=shoot Fe accumulation at anthesis.

$$\begin{array}{c} 2)\text{ Pre-anthesis Fe accumulation}\left(\%\right)\\ =\left(\text{shoot Fe accumulation at anthesis}/\text{shoot Fe accumulation at maturity}\right)\times 100. \end{array}$$

$$\begin{array}{c} 3)\text{Post-anthesis Fe uptake}(\text{kg}/\text{ha})\\ =\text{shoot Fe accumulation at maturity}-\text{shoot Fe accumulation at anthesis.} \end{array}$$

$$\begin{array}{c} 4)\text{ Post-anthesis Fe accumulation}\left(\%\right)\\ =\left(\text{shoot Fe accumulation at post}-\text{anthesis}/\text{shoot Fe accumulation at maturity}\right)\times 100. \end{array}$$

### 2.6 Statistical analysis

Two factor factorial (CRD) was the statistical method used along with ‘GRAPES software (Gopinath et al., 2021). Statistical difference is given in terms of critical difference (CD) values in all the tables.

## 3 Results

### 3.1 Plant growth and yield parameters

#### 3.1.1 Number of tillers and productive tillers

The plants in the OTC had the mean highest number of tillers (17.91/plant), followed by plants in ambient CO_2_ (14.83/plant). The maximum number of tillers was produced in PGPR-treated plants (21.66/plant), followed by AMF- and *Azolla*-treated plants under elevated CO_2_. Control plants under aCO_2_ and eCO_2_ recorded the lowest number of tillers, with 10.66 and 12.66 tillers/plant, respectively. The plants produced an average of 13.16 productive tillers under elevated CO_2_ and 10 productive tillers under ambient CO_2_. The highest number of productive tillers was produced in AMF-treated plants (16.33/plant) under eCO_2_ and lowest number was produced in aCO_2_ control plants, with 5.66 productive tillers. The results are shown in **Table 1**.

**Table 1:** Impact of biofertilisers on tillers and productive tillers in the rice variety Uma under ambient and elevated CO_2_ conditions.

Treatments	Number of tillers at the active tillering stage			Number of productive tillers at harvest		
	Ambient CO_2_	Elevated CO_2_	Mean (T)	Ambient CO_2_	Elevated CO_2_	Mean (T)
Control	10.66^f^	12.66^e^	11.66	5.66^g^	7.33^f^	6.50
*Azolla*	15.66^d^	17.66^c^	16.66	9.66^e^	14.00^c^	11.83
PGPR	16.00^d^	21.66^a^	18.83	12.00^d^	15.00^b^	13.50
AMF	17.00^cd^	19.66^b^	18.33	12.66^d^	16.33^a^	14.50
Mean (C)	14.83	17.91		10.00	13.16	
SE (m) (±)	T=0.323, C=0.228, T×C=0.456			T=0.236, C=0.167, T×C=0.33		
CD (0.05)	T=0.968, C=0.684, T×C=1.368			T=0.707, C=0.500, T×C=0.99		

Small letters mean significant levels.

#### 3.1.2 Dry matter production

The plants grown in elevated CO_2_ produced more dry matter (73.05 g/plant) than those grown in ambient conditions (69.74 g/plant). The application of different biofertilisers led to an overall increase in the production of dry matter by AMF-treated plants (89.13 g/plant under eCO_2_ and 83.83 g/plant under ambient CO_2_). Control plants had lower values in ambient CO_2_ (45.04g/plant) and eCO2 (49.18 g/plant). AMF-treated plants produced the mean highest dry matter (86.48 g/plant) and control plants produced the least (47.11 g/plant). The results are shown in **Table 2**, **Figure 1**.

**Table 2:** Impact of biofertilisers on dry matter production and straw yield in the rice variety Uma under ambient and elevated CO_2_ conditions.

Treatments	Dry matter production (g/plant)			Straw yield (g/plant)		
	Ambient CO_2_	Elevated CO_2_	Mean (T)	Ambient CO_2_	Elevated CO_2_	Mean (T)
Control	45.04^f^	49.18^e^	47.11	24.49^f^	28.74^e^	26.61
*Azolla*	67.93^d^	68.96^d^	68.44	32.60^d^	36.52^c^	34.56
PGPR	82.17^c^	84.92^b^	83.54	36.43^c^	41.43^a^	38.93
AMF	83.83^bc^	89.13^a^	86.48	38.78^b^	41.09^a^	39.94
Mean (C)	69.74	73.05		33.07	36.94	
SE (m) (±)	T=0.495, C=0.35, T×C=0.7			T=0.296, C=0.21, T×C=0.419		
CD (0.05)	T=1.485, C=1.05, T×C=2.1			T=0.888, C=0.628, T×C=1.256		

Small letters mean significant levels.

#### 3.1.3 Straw yield

The mean straw yields of plants in open conditions and the OTC were 33.07 g/plant and 36.94 g/plant, respectively. Control plants had the lowest straw yields (28.74 g/plant and 24.49g/plant in elevated and ambient CO_2_ levels, respectively). The straw yields of both PGPR- and AMF-treated plants were on par under elevated CO_2_, with significantly higher values of 41.43 and 41.09 g/plant, respectively. AMF-treated plants produced the highest mean straw yield (39.94 g/plant) and control plants produced the lowest straw yield (26.61 g/plant). Results are shown in **Table 2**, **Figure 1**.

#### 3.1.4 Number of filled grains per panicle

Under elevated CO_2_, there was a mean total of 75 filled grains per panicle, compared with 108.66 grains per panicle in ambient conditions. With counts of 113.33, 110.00, 108.66, and 102.66 grains/panicle in plants treated with AMF, PGPR, *Azolla* and control plants, respectively, more grains were filled under ambient CO_2_. Grains per panicle were highest in PGPR-treated plants under aCO_2_ (113.33) and lowest in control plants (eCO_2_), with 65 grains per panicle. Results are shown in **Table 3**.

**Table 3:** Impact of biofertilisers on grains per panicle and grain yield in the rice variety Uma under ambient and elevated CO_2_ conditions.

Treatments	No. of filled grains/panicle			Grain yield (g/plant)		
	Ambient CO_2_	Elevated CO_2_	Mean (T)	Ambient CO_2_	Elevated CO_2_	Mean (T)
Control	102.66^c^	65.00^f^	83.83	14.43^g^	10.36^h^	12.39
*Azolla*	108.66^b^	75.00^e^	91.83	25.97^e^	23.59^f^	24.78
PGPR	110.00^b^	79.66^d^	94.83	33.77^b^	30.05^d^	31.91
AMF	113.33^a^	80.33^d^	96.83	37.60^a^	32.65^c^	35.13
Mean (C)	108.66	75.00		27.94	24.16	
SE (m) (±)	T=0.589, C=0.417, T×C=0.833			T=0.217, C=0.154, T×C=0.307		
CD (0.05)	T=1.767, C=1.249, T×C=2.498			T=0.651, C=0.461, T×C=0.921		

Small letters mean significant levels.

#### 3.1.5 Grain yield

Compared with ambient condition plants (27.99 g/plant), elevated CO_2_ condition plants (24.16 g/plant) showed a drop in grain yield. The highest grain yield was recorded in plants treated with AMF under aCO_2_ (37.6 g/plant) and the lowest grain yield was recorded in eCO_2_ control plants (10.36 g/plant). AMF-treated plants in ambient conditions yielded more with 37.60 g/plant and produced 32.65 g/plant under eCO_2_. Control plants, *Azolla*-, and PGPR-treated plants produced mean yields of 12.39 g/plant, 24.78 g/plant, and 31.91 g/plant, respectively; the maximum yield was recorded in AMF-treated plants (35.13 g/plant). Results are shown in **Table 3**, **Figure 1**.

### 3.2 Effect of biofertilisers on the quality parameters of rice grains under ambient COand elevated CO_2_.

#### 3.2.1 Carbohydrate content

Elevated CO_2_ had a significant impact on the carbohydrate content of grains. In control plants, grain carbohydrate content was 706 mg g^-1^ in eCO_2_ grown plants compared with 638.2mg g^-1^ in plants grown in ambient conditions. The mean carbohydrate content in grains under elevated CO_2_ was 766.29 mg g^-1^, which was 10.5% higher than ambient CO_2_ plants. The data are presented in **Table 4**, **Figure 2**.

**Table 4:** Impact of biofertilisers on the quality parameters of grains in the rice variety Uma under ambient and elevated CO_2_ conditions.

Treatments	Quality parameters								
	Carbohydrate content (mg g^-1^)			Protein content (mg g^-1^)			Amylose content (%)		
	aCO_2_	eCO_2_	Mean (T)	aCO_2_	eCO_2_	Mean (T)	aCO_2_	eCO_2_	Mean (T)
Control	638.20^f^	706.40^d^	672.30	6.92^d^	6.06^e^	6.49	20.16	17.81	18.99
*Azolla*	686.30^e^	738.26^b^	712.28	7.61^b^	6.81^d^	7.21	21.27	19.95	20.61
PGPR	725.66^c^	811.15^a^	768.41	7.97^a^	7.41^c^	7.69	22.13	21.14	21.63
AMF	722.63^c^	809.36^a^	766.00	7.72^b^	6.93^d^	7.33	22.76	21.09	21.92
Mean (C)	693.20	766.29		7.56	6.80		21.58	20.00	
SE (m) (±)	T=2.201, C=1.556, T×C=3.112			T=0.032, C=0.022, T×C=0.045			T=0.194, C=0.137, T×C=0.275		
CD (0.05)	T=6.598, C=4.666, T×C=9.331			T=0.095, C=0.067, T×C=0.134			T=0.583, C=0.412, T×C=NS		

Small letters mean significant levels.

Biofertilisers had a significant impact on the carbohydrate content of grains under both ambient and elevated CO_2_ conditions. PGPR-treated plants recorded the highest value (725.66 mg g^-1^) for grain carbohydrate content, which was on a par with AMF-treated plants under ambient conditions (722.63 mg g-1). The same trend was observed under elevated CO_2_ in both PGPR- and AMF-treated plants, which had the highest values (811.15mg g^-1^ and 809.36mg g^-1^, respectively) by a significant margin.

#### 3.2.2 Protein content

Protein content in grains significantly dropped under elevated CO_2_ compared with ambient conditions. In control plants, the protein contents of grains were 6.92 mg g^-1^ (aCO_2_) and 6.06 mg g^-1^ (eCO_2_). The mean protein contents in grains under eCO_2_ and ambient conditions were 6.8 mg g-1 and 7.56 mg g^-1^, respectively. The data are presented in **Table 4**, **Figure 2**. Biofertiliser application significantly increased protein content in grains under both the CO_2_ conditions. Protein content in PGPR-treated plant grains under aCO_2_ was high (7.97 mg g^-1^); protein content was 7.41 mg g^-1^ under eCO_2_.

#### 3.2.3 Amylose content (%)

The data regarding amylose content in grains are shown in **Table 4**. Under elevated CO_2_, grain amylose content was reduced in control plants (17.81 mg g^-1^) compared with ambient conditions (20.16 mg g^-1^). The mean amylose content in grains was 21.58 mg g^-1^ and 20 mg g^-1^ under ambient and elevated CO_2_ conditions, respectively. Amylose content of grains was increased by biofertiliser treatment. AMF-treated plants had higher amylose content (22.76mg g^-1^ and 21.09mg g^-1^ under ambient and elevated CO_2_ conditions respectively).

### 3.3 Iron uptake and translocation characters

#### 3.3.1 Pre-anthesis stage: Fe content, uptake, and accumulation

##### 3.3.1.1 Iron content in roots and shoots

Elevated CO_2_ decreased Fe content in roots and shoots of rice at the pre-anthesis stage. For control plants, Fe content was low under eCO_2_ (48.38 ppm) and high in ambient conditions (52.83 ppm). Mean root Fe content was decreased under eCO_2_ (54.63 ppm) compared with ambient conditions (60.09 ppm). For shoots, the Fe content was 35.75 ppm under ambient conditions and 31.77 ppm under eCO_2_. This decrease in Fe content was increased by biofertilisers.

PGPR-treated plants had Fe content values in both roots and shoots. In roots, PGPR under aCO_2_ recorded high Fe content (65.8 ppm): Fe content in shoots was 39.11ppm. The root and shoot Fe contents in PGPR-treated plants (eCO_2_) were higher than in the control plants under ambient conditions. The lowest Fe content values were recorded in control plants under eCO_2_, with 48.38 ppm in roots and 27.21 ppm in shoots. The results are shown in **Table 5**.

**Table 5:** Impact of biofertilisers on the iron content of roots and shoots at the pre-anthesis stage in the rice variety Uma under ambient and elevated CO_2_ conditions.

Treatments	Pre-anthesis stage Fe content (ppm)					
	Roots			Shoots		
	Ambient CO_2_	Elevated CO_2_	Mean (T)	Ambient CO_2_	Elevated CO_2_	Mean (T)
Control	52.83	48.38	50.61	30.54	27.21	28.87
*Azolla*	58.31	53.25	55.78	35.13	30.10	32.61
PGPR	65.80	59.21	62.51	39.11	34.58	36.84
AMF	63.43	57.67	60.55	38.24	35.19	36.72
Mean (C)	60.09	54.63		35.75	31.77	
SE (m) (±)	T=0.311, C=0.22, T×C=0.439			T=0.282, C=0.199, T×C=0.398		
CD (0.05)	T=0.931, C=0.659, T×C=NS			T=0.844, C=0.597, T×C=NS		

##### 3.3.1.2 Iron uptake and accumulation

The lowest value for iron uptake was found in control plants (eCO_2_) with 0.17 kg/ha. Mean Fe uptake was significantly higher in the open CO_2_ condition (0.25 kg/ha) than in elevated CO_2_ (0.23 kg/ha). The uptake of iron in rice under different biofertilisers treatments was significantly higher in PGPR-treated plants (0.30 kg/ha [aCO_2]_) than with *Azolla* and AMF treatments. The mean Fe uptake was greater in PGPR-treated plants (0.29 kg/ha) and lower in control plants (0.18 kg/ha). The data are shown in **Table 6**.

**Table 6:** Impact of biofertilisers on iron uptake and accumulation at the pre-anthesis stage in the rice variety Uma under ambient and elevated CO_2_ conditions.

Treatments	Pre-anthesis stage					
	Fe uptake (kg/ha)			Fe accumulation (%)		
	Ambient CO_2_	Elevated CO_2_	Mean (T)	Ambient CO_2_	Elevated CO_2_	Mean (T)
Control	0.19	0.17	0.18	65.39	62.62	64.01
*Azolla*	0.21	0.20	0.21	68.37	67.86	68.12
PGPR	0.30	0.28	0.29	72.63	71.00	71.81
AMF	0.28	0.27	0.27	69.79	68.91	69.35
Mean (C)	0.25	0.23		69.05	67.60	
SE (m) (±)	T=0.002, C=0.002, T×C=0.003			T=0.405, C=0.286, T×C=0.572		
CD (0.05)	T=0.006, C=0.005, T×C=NS			T=1.213, C=0.858, T×C=NS		

Fe accumulation in control plants was also higher under ambient conditions (65.39 ppm) than under elevated CO_2_ (62.62 ppm). Fe accumulation during the pre-anthesis stage was higher in PGPR-treated plants (71.81%). Higher accumulation was recorded in PGPR -treated plants (aCO_2_) (72.63%).

#### 3.3.2 Post-anthesis stage: Fe uptake and accumulation

For the post-anthesis stage, a higher uptake of Fe was found in ambient plants (0.35kg/ha) than in elevated CO_2_ condition plants (0.32kg/ha). Fe uptake and accumulation were significantly increased by various biofertilisers. The mean Fe uptake was highest in ambient PGPR-treated plants (0.42 kg/ha) and lowest in control plants under elevated CO_2_ (0.24 kg/ha).

The percentage of Fe accumulation was non-significant and lower in the elevated CO_2_ conditions when compared to control plants in ambient conditions. Eventhough, it was non-significant, the %Fe accumulation was less under elevated CO_2_ condition plants when compared to ambient condition plants. Mean higher and lower values of 50.28% and 43.07% were recorded in PGPR and control plants, respectively. The highest Fe accumulation value was recorded in PGPR-treated plants under ambient conditions with 50.63 ppm (49.93 ppm under eCO_2_). The results pertaining to Fe uptake and accumulation at the post-anthesis stage are shown in **Table 7**.

**Table 7:** Impact of biofertilisers on iron uptake and accumulation at the post-anthesis stage in the rice variety Uma under ambient and elevated CO_2_ conditions.

Treatments	Post-anthesis stage					
	Fe uptake (kg/ha)			Fe accumulation (%)		
	Ambient CO_2_	Elevated CO_2_	Mean (T)	Ambient CO_2_	Elevated CO_2_	Mean (T)
Control	0.27	0.24	0.26	43.79	42.35	43.07
*Azolla*	0.31	0.28	0.29	47.67	46.09	46.88
PGPR	0.42	0.38	0.40	50.63	49.93	50.28
AMF	0.39	0.38	0.39	47.56	48.91	48.24
Mean (C)	0.35	0.32		47.41	46.82	
SE (m) (±)	T=0.004, C=0.003, T×C=0.006			T=0.625, C=0.442, T×C=0.884		
CD (0.05)	T=0.013, C=0.009, T×C=NS			T=1.875, C=NS, T×C=NS		

#### 3.3.3 Iron partitioning at harvest

##### 3.3.3.1 Roots and shoots

Iron portioning into roots and shoots at harvest (**Table 8**) was higher under ambient CO_2_ conditions than under eCO_2_. In roots, Fe content was lower in OTC plants (60.26 ppm) than in ambient plants (65.37 ppm). Fe content was higher in PGPR-treated plants (aCO_2_) (73.68 ppm) and lower in control plants (eCO_2_) (55.07 ppm) **Figure 3a**.

**Table 8:** Impact of biofertilisers on iron partitioning at harvest in the rice variety Uma under ambient and elevated CO_2_ conditions.

Treatments	Iron content (ppm)					
	Roots			Shoots		
	Ambient CO_2_	Elevated CO_2_	Mean (T)	Ambient CO_2_	Elevated CO_2_	Mean (T)
Control	59.59	55.07	57.33	37.33	33.99	35.66
*Azolla*	61.67	57.18	59.42	41.91	37.02	39.46
PGPR	73.68	67.81	70.74	46.07	41.36	43.72
AMF	66.54	60.98	63.76	42.81	38.20	43.50
Mean (C)	65.37	60.26		42.03	37.64	
SE (m) (±)	T=0.529, C=0.374, T×C=0.748			T=0.266, C=0.188, T×C=0.377		
CD (0.05)	T=1.585, C=1.121, T×C=NS			T=0.779, C=0.565, T×C=NS		

In aCO_2_ and eCO_2_, Fe contents were 42.03ppm and 37.64ppm, respectively, in shoots. Shoot Fe content at harvest was higher in PGPR-treated plants (46.07 ppm) and lower in control plants (33.99 ppm) under ambient and OTC conditions, respectively. Mean shoot Fe content was highest in PGPR-treated plants (43.72 ppm), followed by AMF-treated plants (43.50 ppm) and *Azolla*-treated plants (39.46 ppm), and lowest in control plants (35.66 ppm).

##### 3.3.3.2 Grains and bran

Fe contents in grains, bran, and polished rice significantly decreased under elevated CO_2_ when compared with ambient conditions **Figure 3B**. A comparatively high Fe content was recorded in grains from ambient CO_2_ plants (17.16 ppm) but was lower under eCO_2_ (13.76 ppm). Grain iron content was highest in PGPR-treated plants (20.69 ppm) under ambient CO_2_ and lowest in control plants under eCO_2_ (9.89 ppm). Mean Fe concentration was highest in PGPR-treated plants (19.27 ppm), followed by AMF-treated plants (17.46 ppm) and *Azolla*-treated plants (14.18 ppm), and lowest in control plants (10.93 ppm).

Rice bran Fe content in open condition plants (130.9 ppm) was higher than in the OTC condition plants (118.8 ppm). Bran Fe content increased after biofertiliser treatment. Rice bran Fe content was highest in PGPR-treated plants under ambient conditions (143.2 ppm) and lowest in control plants under eCO_2_ (104.5 ppm).

Ambient CO_2_ plants had a higher mean Fe content in polished rice (3.72 ppm) than under eCO_2_ (3.22 ppm). For polished rice grains, Fe content was higher in PGPR-treated plants (4.24 ppm) under aCO_2_ and lower in control plants (2.35 ppm) under eCO_2_. Mean Fe content was higher in PGPR-treated plants (4.02 ppm) and lower in control plants (2.62 ppm). The results related to grains and bran and polished rice Fe content are presented in **Table 9**.

**Table 9:** Impact of biofertilisers on iron content in grains and rice bran in the rice variety Uma under ambient and elevated CO_2_ conditions.

Treatments	Fe content (ppm)								
	Grains			Bran			Polished rice		
	aCO_2_	eCO_2_	Mean (T)	aCO_2_	eCO_2_	Mean (T)	aCO_2_	eCO_2_	Mean (T)
Control	11.97^e^	9.89^f^	10.93	117.1	104.5	110.80	2.90	2.35	2.62
*Azolla*	16.62^cd^	11.77^e^	14.18	128.1	113.3	120.70	3.63	3.19	3.41
PGPR	20.69^a^	17.86^c^	19.27	143.2	134.4	138.82	4.24	3.80	4.02
AMF	19.38^b^	15.53^d^	17.46	135.5	123.1	129.33	4.11	3.55	3.83
Mean (C)	17.16	13.76		130.9	118.8		3.72	3.22	
SE (m) (±)	T=0.309, C=0.219, T×C=0.437			T=1.027, C=0.726, T×C=1.452			T=0.02, C=0.014, T×C=0.028		
CD (0.05)	T=0.927, C=0.655, T×C=1.31			T=3.078, C=2.176, T×C=NS			T=0.059, C=0.042, T×C=NS		

## 4 Discussion

### 4.1 Plant growth and yield parameters

Elevated CO_2_ caused a 20.76% mean increase in tiller number compared with ambient conditions. Tillers exhibited an approximate 100% increase in number during the active tillering stage in PGPR-treated plants under eCO_2_ compared with control plants in ambient conditions and a 35% increase compared with ambient PGPR-treated plants. The productive tiller number in OTC plants was 31.67% higher than in open condition plants because elevated CO_2_ favours a high photosynthetic rate and resulted in greater shoot, root, and spike production and faster canopy development by increasing leaf and tiller appearance rates and phenology. The production of productive tillers was significantly high in AMF-treated plants under eCO_2_. The growth stimulation of plants grown under eCO_2_ is due to increased photosynthate availability in meristems, which increases the proportion of rapidly dividing cells by stimulating cell division. The non-structural carbohydrates that accumulate in plant tissues under eCO_2_ stimulate the cell cycle and auxin biosynthesis, which increases plant growth and biomass.

For dry matter, a rise in DMP by 6.3% was observed under eCO_2_ compared with ambient conditions. AMF-treated plants significantly increased production under eCO_2_ compared with the control plants. Straw yield in the present study was higher under elevated CO_2_, with an overall increase of 11.7% compared with ambient plants. Straw yield was higher in the plants grown under elevated CO_2_ and in both AMF- and PGPR-treated plants, which were on a par with the eCO_2_ condition. AMF (eCO_2_) had a 67.7% higher straw yield, while PGPR (eCO_2_) had a 69.17% higher straw yield than control plants (aCO_2_). This increase in straw yield and DMP were due to higher plant height, a greater number of tillers, high water-use efficiency of vegetative biomass, and greater accumulation of photosynthates in the shoots of PGPR- and AMF-treated plants under eCO_2_ (Gardi et al., 2021).

An overall decrease in grain number was observed under eCO_2_ (27.7%) compared with ambient plants. This experimental study was in line with Rosalin et al. (2018), whose experiment resulted in increased grain chaffiness (147%) under eCO_2_. An increase in temperature as a result of CO_2_ enrichment would have resulted in poor pollen viability, leading to increased chaffiness. The effects of increased temperature under elevated CO_2_ on rice grain yield and reproductive processes were detrimental (Prasad et al., 2006; Beena et al., 2021; Stephen et al., 2022). A 10.4% increase in the number of filled grains per panicle was observed in AMF-treated plants compared with control plants in ambient conditions, and likewise, there was a 23.5% under eCO_2_.

Control plants under aCO_2_ produced a 39.28% higher yield than eCO_2_ control plants. This reduction in seed yield in OTC plants was due to fewer grains per panicle, which was compensated by the presence of more panicles to an extent. Similar studies were carried out by Jena et al. (2018) in rice and Prasad et al. (2006) in rice and *Sorghum*, who showed that induced temperature decreased pollen germination by 48% under elevated CO_2_. Relative to the ambient control plots, a consistent temperature increase of 2.84°C and 1.83°C was respectively recorded in the air and soil in an OTC chamber (Sun et al., 2013). Elevated CO_2_ decreased pollen germination by 9% at 32/22°C and 36% at 36/26°C, which will lead to a reduction in grain formation.

The mechanism by which PGPR increase growth involves the production of phytohormones, such as auxins and gibberellins, the suppression of deleterious organisms, the activation of phosphate solubilisation, and the promotion of mineral nutrient uptake. Ashrafuzzaman et al. (2009) experimental results suggested that the increased growth of rice seedlings by PGPR is probably due to phosphorus solubilisation and IAA production, which is responsible for cell elongation.

AMF improves phosphorus mobilization and the uptake from the soil into the plant. This increases P in the plant and induces the production of strigolactones, which are signalling components produced by plants and act as endogenous hormones to promote symbiotic interactions between plants and soil microbes. Strigolactones are likely to regulate shoot branching and gravitropism in parallel through auxin transport and biosynthesis; ultimately, these will improve the productive tillers in rice (Courto et al., 2013). The high number of grains per panicle in AMF-treated plants could be due to higher nutrient uptake, especially N, P, and Mg, which could have started at the seedling growth stage, as reported by Wangiyana et al. (2021).

### 4.2 Quality parameters of rice grains

#### 4.2.1 Carbohydrate content

The present study showed that grain carbohydrate content increased by 10.5% under elevated CO_2_ compared with the open condition. High CO_2_ levels lead to increased grain carbohydrates due to a greater production of photosynthates. PGPR- and AMF-treated plants under elevated CO_2_ had 27% more carbohydrates in grains than in those of ambient control plants at harvest. This might be caused by an increase in the translocation of carbohydrates to grains because of increased sink capacity in the grains of PGPR-treated plants. AMF inoculated plant roots become a strong sink for carbohydrates as these fungi can consume up to 20% of the host plant sugars in rice. These results are supported by Panneerselvam et al. (2019) in rice.

#### 4.2.2 Grain protein content

Grain protein content was higher in ambient plants. A 12.3% drop in grain protein content in elevated eCO_2_ plants compared with the open condition is due to a decrease in Rubisco concentration, most likely due to a carbohydrate-dependent decrease in the expression of photosynthetic genes and the downregulation of some transporter proteins under excess CO_2_. The mechanism for protein reduction is dilution by increased concentrations of non-structural carbohydrates (Gifford et al., 2000). This is supported by Goufo et al. (2014) in rice. Grain protein content was 12% higher in ambient PGPR-treated plants than in the control plants (aCO_2_) due to organic acid secretions by PGPR *via* proton pumps through ATPase, which can acidify the rhizosphere, which in turn increases the plant uptake of mineral nutrients, such as Ca, K, Fe, Cu, Mn, and Zn (Swarnalakshmi et al., 2020).

#### 4.2.3 Grain amylose content

Amylose is a starch molecule responsible for the non-gelatinisation of rice grains during cooking, i.e., grains with high amylose (>24%) will be considered as good quality rice grains. In the present experiment, the amylose content was more in AMF-treated rice grains under ambient conditions, which was supported by Tang et al. (2022) in rice and Harish et al. (2022) in maize. Under elevated CO_2_, amylose content decreased by 8% due to the limitation of starch accumulation; a rapid turnover of starch to free sugars in the fast-developing rice grain may explain the decrease of amylose observed by Yang et al. (2007).

### 4.3 Iron uptake and translocation characters

In the present study, the Fe content, accumulation, and uptake were analysed at the pre-anthesis and post-anthesis stages of rice. Pre-anthesis Fe uptake shares a relevance with Fe translocation and post-anthesis with Fe remobilisation characteristics. Translocation is the movement of materials from root to shoot and remobilisation is the movement of Fe from shoot to grains. The degree of Fe accumulation in grains is dependent upon remobilisation from pre-anthesis shoot accumulation and post-anthesis uptake.

In the present study, Fe content in roots and shoots during the pre-anthesis stage dropped by 10–12.5% under eCO_2_ compared with aCO_2_. Pre-anthesis Fe uptake in control plants dropped by 11.76% and Fe accumulation dropped by 4.4% under eCO_2_. This decrease in Fe translocation was increased to an extent by PGPR treatment. The PGPR-treated plants showed an increase in root Fe content by 24.5% and 22.38% compared with control plants in ambient and elevated CO_2_ conditions, respectively. Likewise, shoot Fe content increased by 28% and 27.08% with PGPR treatment compared with control plants under aCO_2_ and eCO_2_.

Fe uptake during the pre-anthesis stage, i.e., the translocation of Fe from roots to shoots, increased due to PGPR application by 57.89% under aCO_2_ and 64.7% under eCO_2_ compared with control plants. Additionally, the Fe accumulation percentage was increased by PGPR activity under both the CO_2_ conditions. This increased Fe uptake is due to the enhanced activity of PGPR under eCO_2_. PGPR-treated plants accumulated more Fe (72.63%) than control plants (65.39%) under aCO_2_. Increases in Fe accumulation of 71% (PGPR-treated plants) and 62.62% (control plants) were recorded under elevated CO_2_. On average, PGPR-treated plants under eCO_2_ increased Fe uptake by 47.36% compared with control plants in ambient conditions.

For the post-anthesis stage, Fe uptake increased compared with the pre-anthesis stage; possibly due to the increased Fe remobilisation from shoots to grains. The Fe accumulation percentage decreased during the post-anthesis stage due to the remobilisation of pre-anthesis accumulated Fe into grains. This remobilised Fe will accumulate in grains. During the post-anthesis stage also, there was a drop in Fe uptake in control plants under eCO_2_ by 12.5%. Fe uptake increased in PGPR-treated plants by 55% and 58% compared with control plants under ambient and elevated CO_2_ conditions, respectively. Overall, Fe uptake increased by 40.7% in eCO2 PGPR-treated plants compared with aCO_2_ control plants. These results are supported by Liu et al. (2019) in wheat Zn homeostasis, in which an increase in Zn uptake was observed during the post-anthesis stage compared with the pre-anthesis stage, and Zn accumulation decreased during the post-anthesis stage due to remobilisation.

Although Fe content in shoots and roots decreased under eCO_2_, Fe accumulation increased in shoots due to an increase in plant biomass under eCO2. This increased biomass in eCO_2_ plants compensated for the decreased Fe content in plants to an extent. There was a 4.4% and 3.4% drop in Fe accumulation in control plants under eCO_2_ at the pre-anthesis and post-anthesis stages, respectively. The percentage of Fe content in the polished rice depends upon the Fe content in grains. The partitioning of Fe will be more into polished rice if the grains have a greater Fe content.

At harvest, Fe content dropped under elevated CO_2_ compared with ambient CO_2_ by 8.47% and 11.66% in roots and straw, respectively. The iron partitioning at harvest is greatest in roots, followed by straw and grains. Grain Fe content in control plants under eCO_2_ dropped by 21%. This decreased Fe content can be increased by PGPR by 80.5% under elevated CO_2_ and by 72.8% under aCO_2_ compared with control plants under the respective CO_2_ conditions.

**Figure 4:**
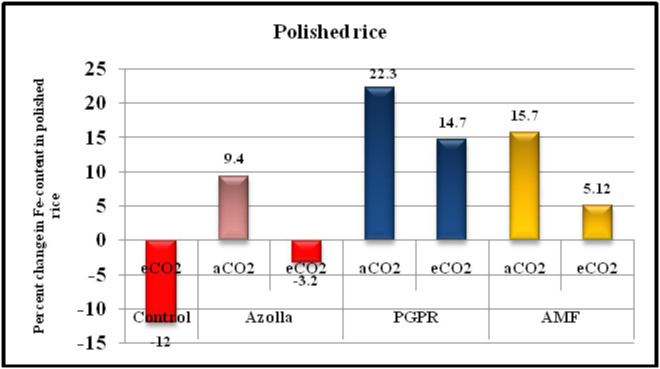
Percentage change in Fe-content of polished rice in comparison with control plants under ambient condition.

When seeds were milled, the Fe concentration drastically decreased because the Fe rich portions of the seeds were eliminated. Supporting articles include Shylaraj et al. (2018) and Trinidad et al., 2009. The Fe content in grains was partitioned more into bran than into the polished rice. The rice bran Fe content in control plants decreased by 12% and in polished rice by 23.4% under elevated CO_2_. This highlights the importance of the consumption of whole grains. Biofertilisers were found to play a significant role in the present scenario. The application of biofertilisers enhanced the Fe status in rice grains. The most effective biofertiliser in this regard were PGPR. The drop in Fe content was increased by 28.6% and 61.7% with PGPR treatment in bran and polished rice, respectively, under elevated CO_2_ and by 22.3% and 46.2% under ambient conditions compared with control plants under the respective CO_2_ conditions (**Figure 4**).

The effects of CO_2_ enrichment generally resulted in a “dilution” of nutrients in stems. This was particularly noticeable during anthesis and the grain filling stages and was most likely caused by the build-up of non-structural carbohydrates, which also resulted in a drop in the concentration of Ca, S, and Fe (Fangmeier et al., 1999) in wheat. The complicated effects of CO_2_ enrichment on nutrient intake, distribution, and redistribution were evident from the results of this programme. CO_2_ enrichment affects crop growth and quality in a number of ways from an agricultural standpoint.

Lower absorption and translocation were key factors underlying the lower elemental content of Fe under elevated CO_2_. During re-translocation, accumulated elements were transported with carbohydrates in phloem sap. Therefore, in elevated CO_2_ conditions, it affects the elemental re-translocation *via* carbohydrate translocation. Fe-regulated transporter-like protein, *OsZIP5*, plays a key role in Fe deficiency and is controlled at a transcriptional level in rice, and its expression is reduced by almost half under elevated CO_2_. Elevated CO_2_ also negatively regulates the expression of the rice nicotianamine synthase gene (*OsNAS3*), which is involved in the transport of iron into developing grains (Ujiie et al., 2019). Seneweera and Conroy (1997) reported a decrease of 17% for Fe in rice grains grown under elevated CO_2_. Increasing the concentration of CO_2_ in the atmosphere can increase the accumulation of carbohydrates in plants, which can lead to a “dilution effect”, resulting in a lower Fe content.

PGPR play an important role in the regulation of Fe uptake and accumulation under its limited availability by accumulating and exuding organic acids, phenolic compounds, and siderophores and enhancing FCR enzyme activities. Siderophores are low molecular weight compounds that, when released into soil, chelate with Fe^+3^ and reduce it to Fe^+2^. This increases the availability of iron and uptake into plants *via* specific transport systems. PGPR can activate the genes associated with Fe deficiency, such as the ferric chelate reductase gene (*FCR*) and the Fe^2+^ transporter gene (*IRT1*). Microorganisms have evolved specialised mechanisms for the assimilation of Fe, including the production of siderophores, which transport Fe into their cells.

The *Bacillus* strains that were present in PGPR inoculum were very strong solubilisers of Fe, which increased the availability of Fe^+2^ in the soil and to plants. These *Bacillus* strains increase mugineic acid biosynthesis, which enhances the Fe chelation capacity and Fe uptake. In a comparative proteomics analysis, PGPR upregulated the Nicotianamine synthase 1 (*NAS1*) gene by 100-fold in rice. PGPR also increased ferritin isoforms (*Fer552* and *Fer768*), which increased Fe storage in grains (Alberton et al., 2020). This mechanism of Fe uptake and storage increased shoot and root biomass and increased grain sink capacity through PGPR activity can increase the Fe content and accumulation in plants. PGPR-treated plants increase translocation and remobilisation in rice plants through increased uptake and accumulation (Sharma et al., 2013). Chen et al. showed an increase in rice plant growth and yield through the combined application of PGPR and AMF due to a significant increase in the availability of nutrients. This group also highlighted the ability of soil microbes, such as PGPR, to support material circulation, nutrient transformation, and other soil biochemical processes that are crucial for the soil ecosystem.

## 5 Conclusion

Diminishing rice quality with the increasing concentration of CO_2_ is a global concern today. Elevated CO_2_ had a positive impact on growth but negatively influenced grain yield due to eCO_2_-associated high temperatures. The response of experimental plants to AMF and PGPR treatments suggest that these biofertilisers can be recommended to overcome the impacts of elevated CO_2_-associated high temperatures and thereby improve plant performance. In the present programme, Fe uptake and translocation were modified in an unfavourable manner under eCO_2_, which was reflected by the lower Fe content in rice grains. All the biofertilisers, *Azolla*, PGPR, and AMF, had a significant impact on grain Fe content under both ambient and elevated CO_2_ conditions. PGPR was found to be the most effective at increasing Fe content in the polished grain as well as in the bran under both conditions. The response of Fe homeostasis by the experimental plants to the application of biofertilisers, especially PGPR, under eCO_2_ strongly suggests the possibility of utilising them for designing Fe management strategies for achieving higher rice yield and quality. As the present study highlights the importance of the consumption of whole grains, it is of paramount importance to organise programmes at all levels of population to generate awareness on the dangers of the consumption of polished rice and also on the health issues being generated due to low intake of micronutrients, which is becoming serious in the changing climatic scenario.

## Data availability statement

The original contributions presented in the study are included in the article/supplementary material. Further inquiries can be directed to the corresponding author.

## Author contributions

RM conceptualized the study. MB performed the work. Data analysis was carried out by MB and RM. Technical guidance and support was provided by RM, MV, SR, KA, and RB. All authors reviewed and approved the manuscript.
